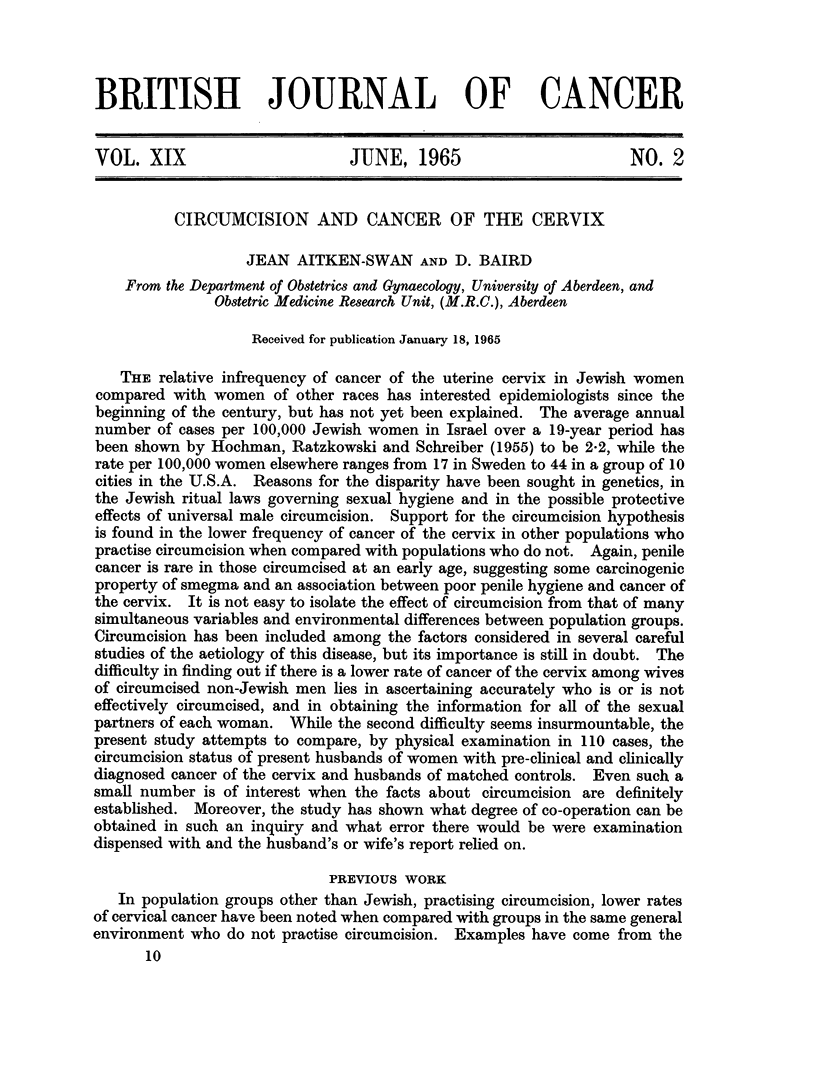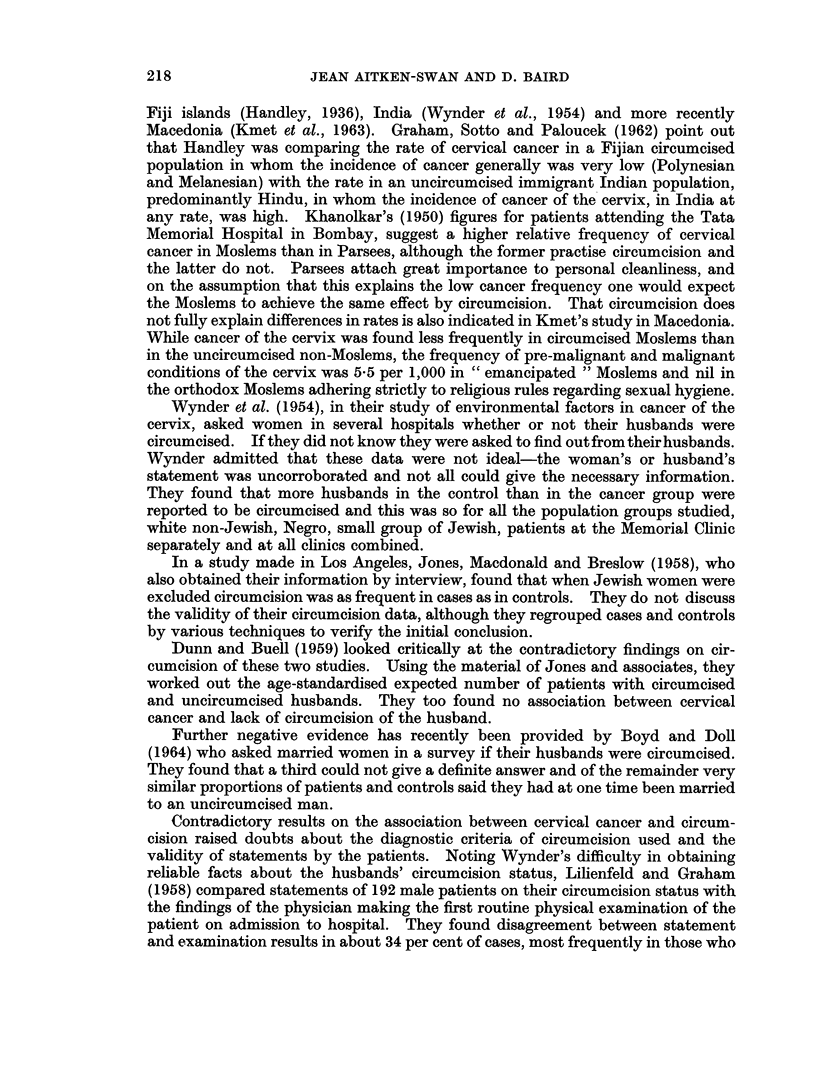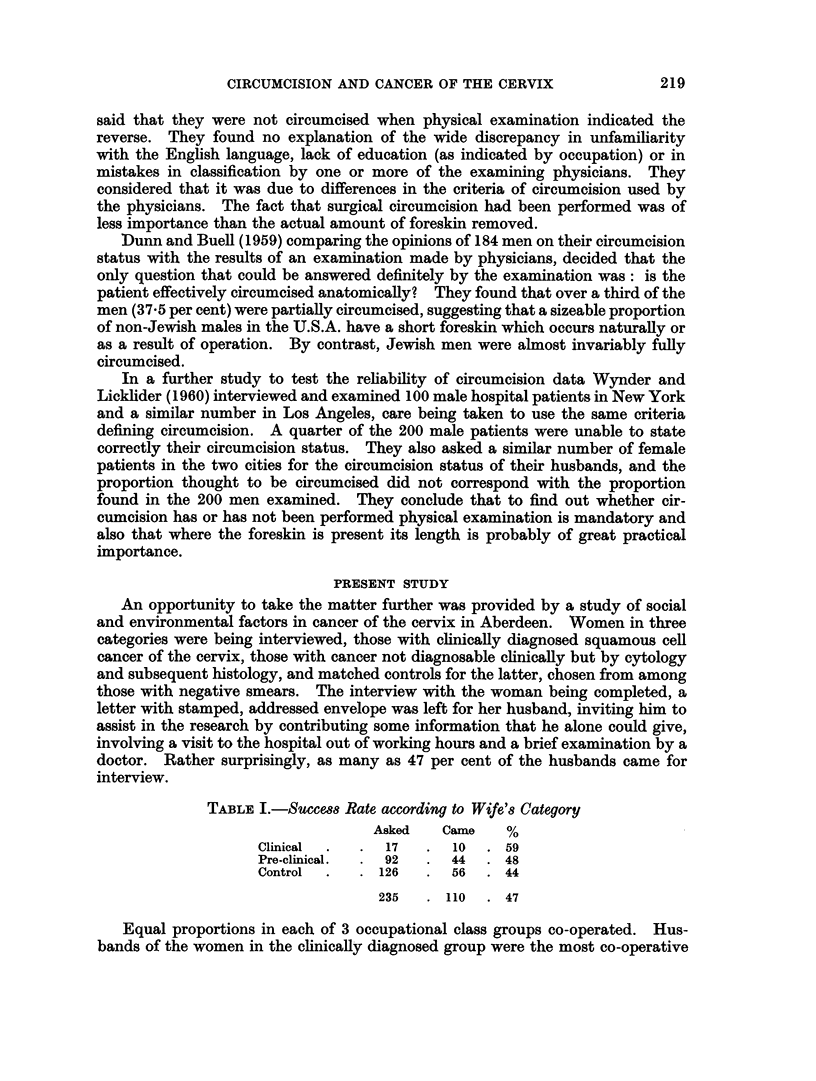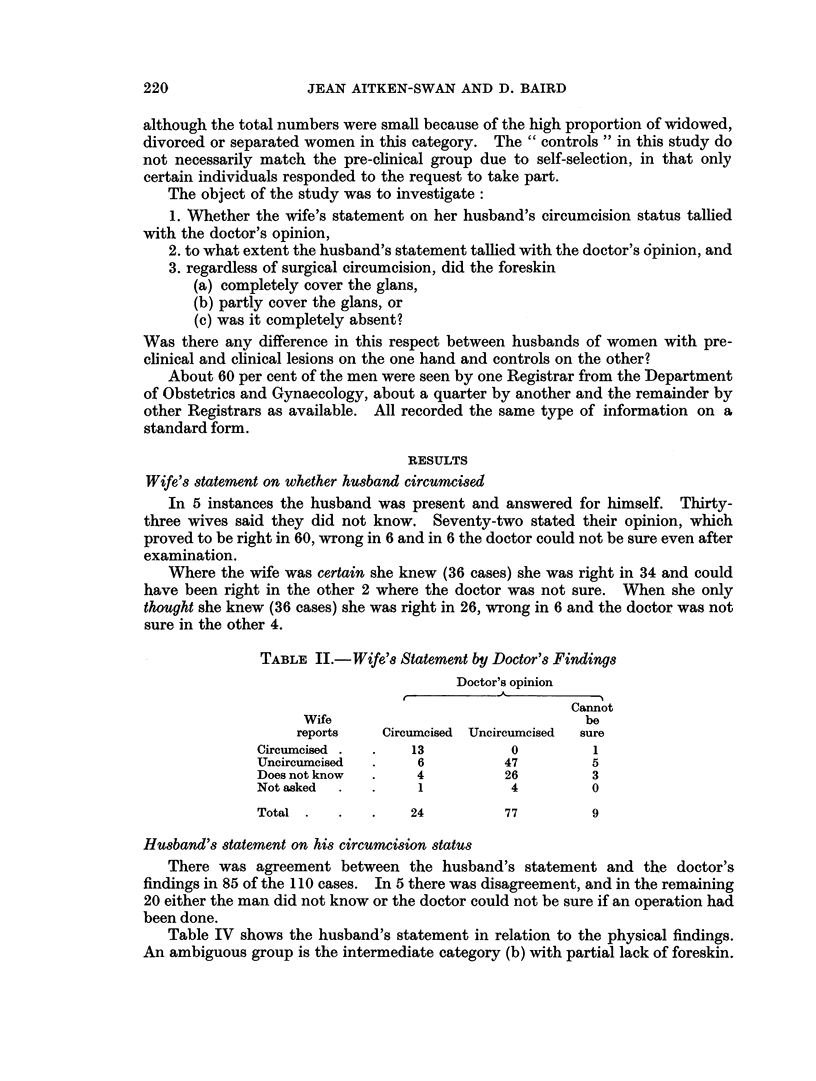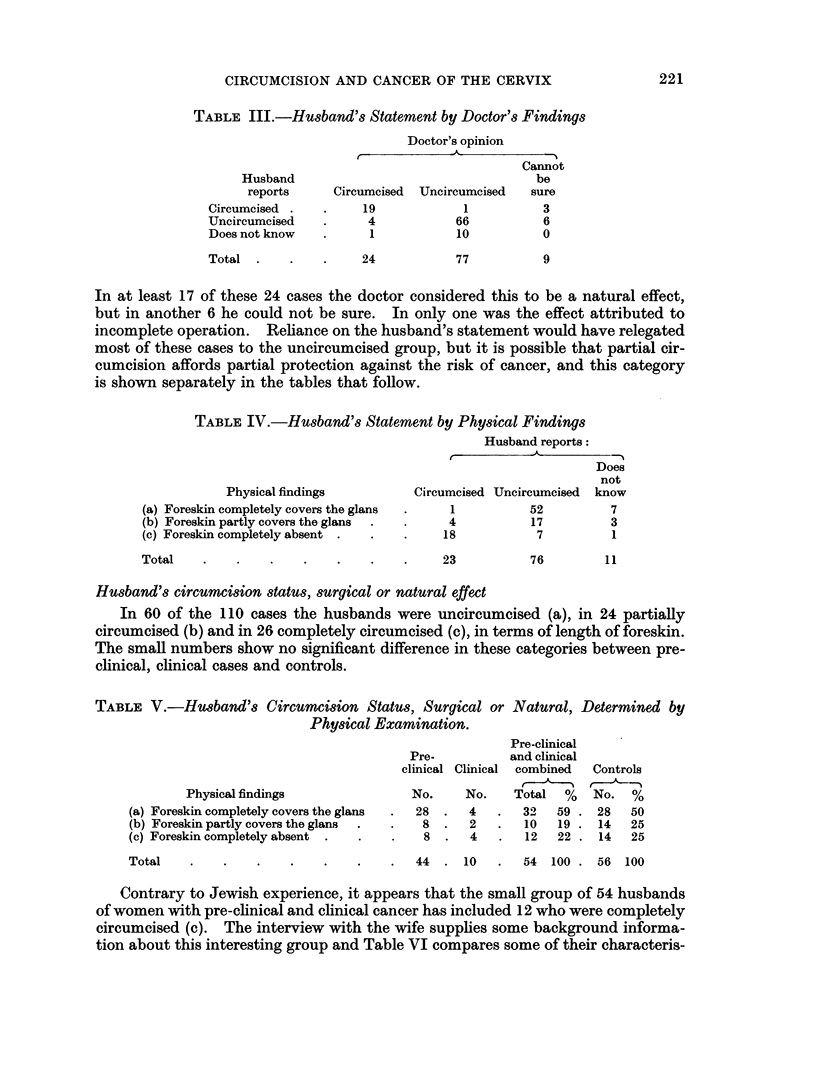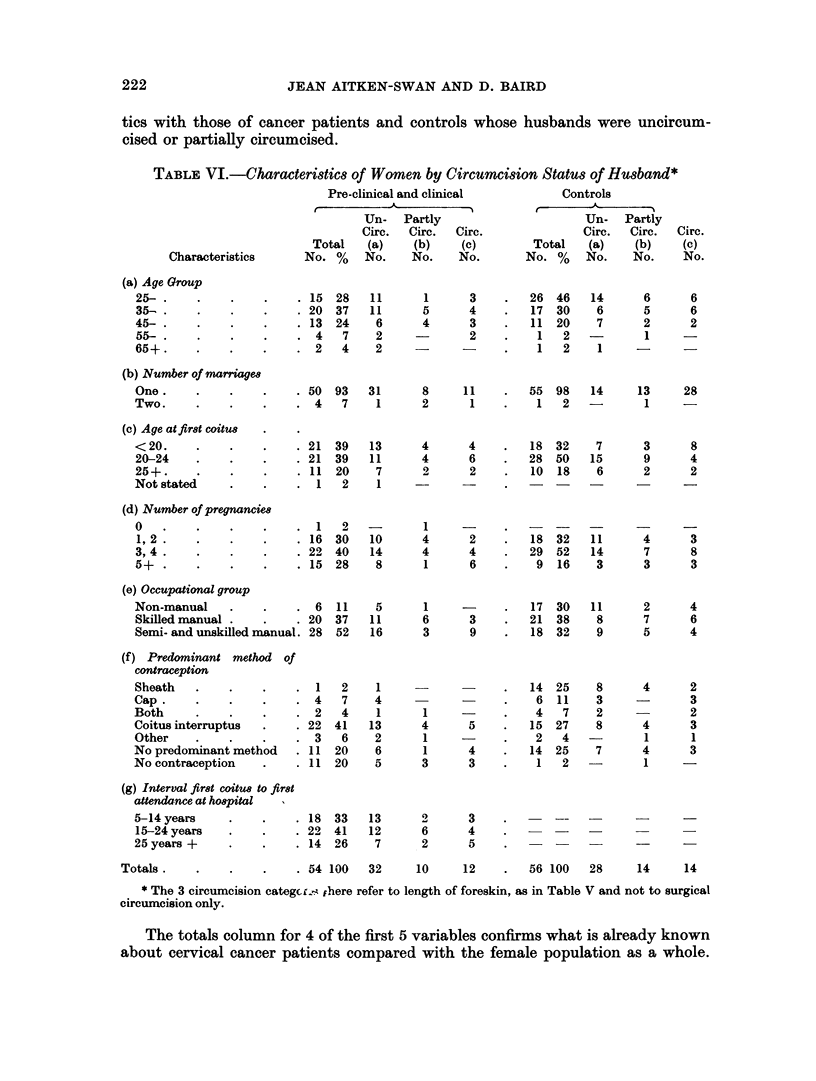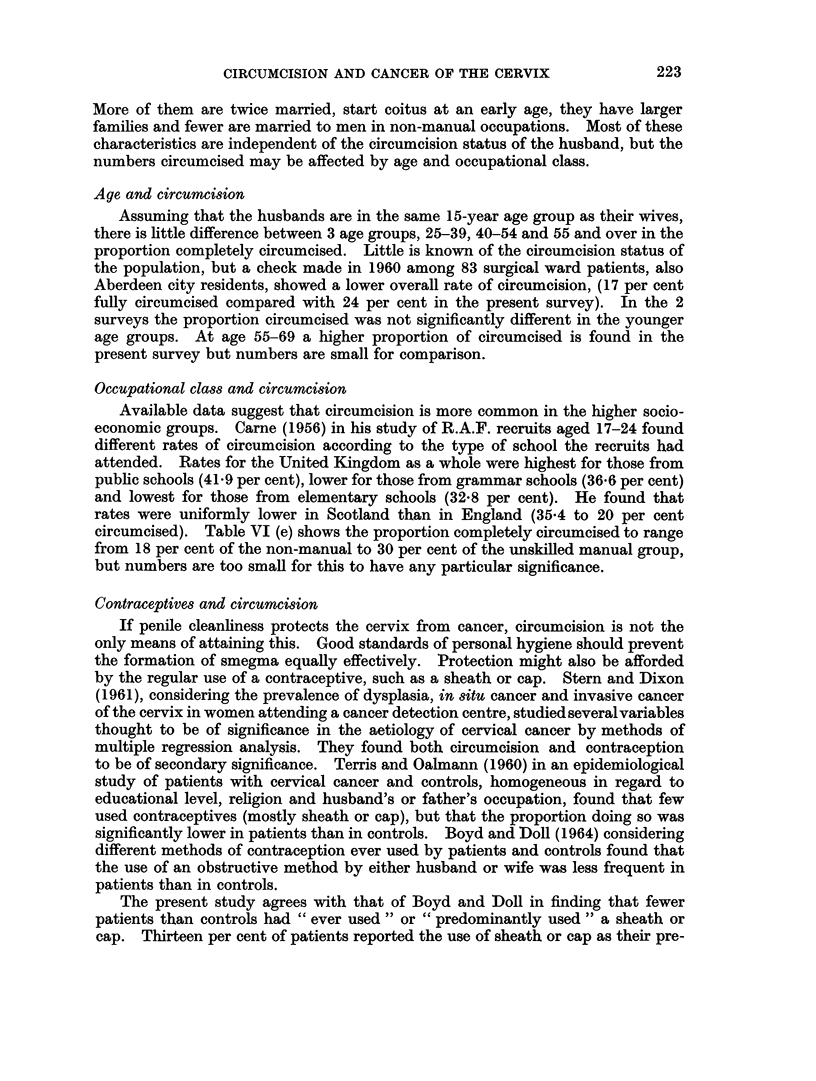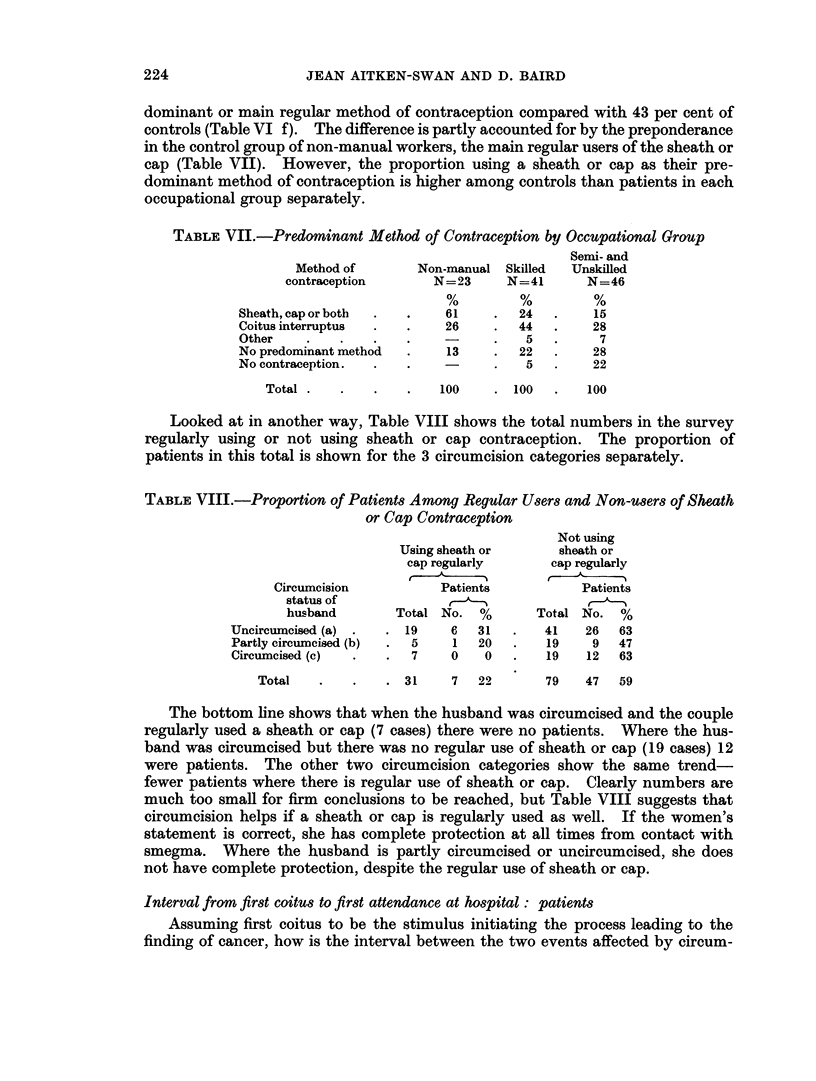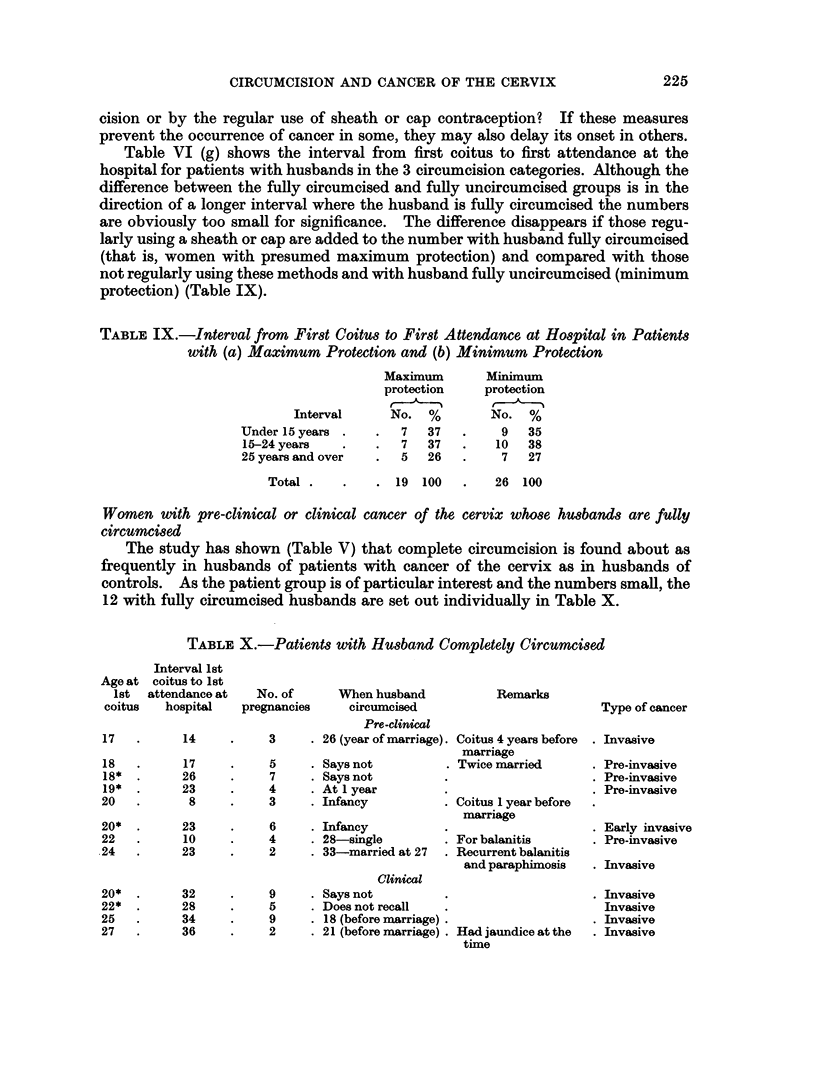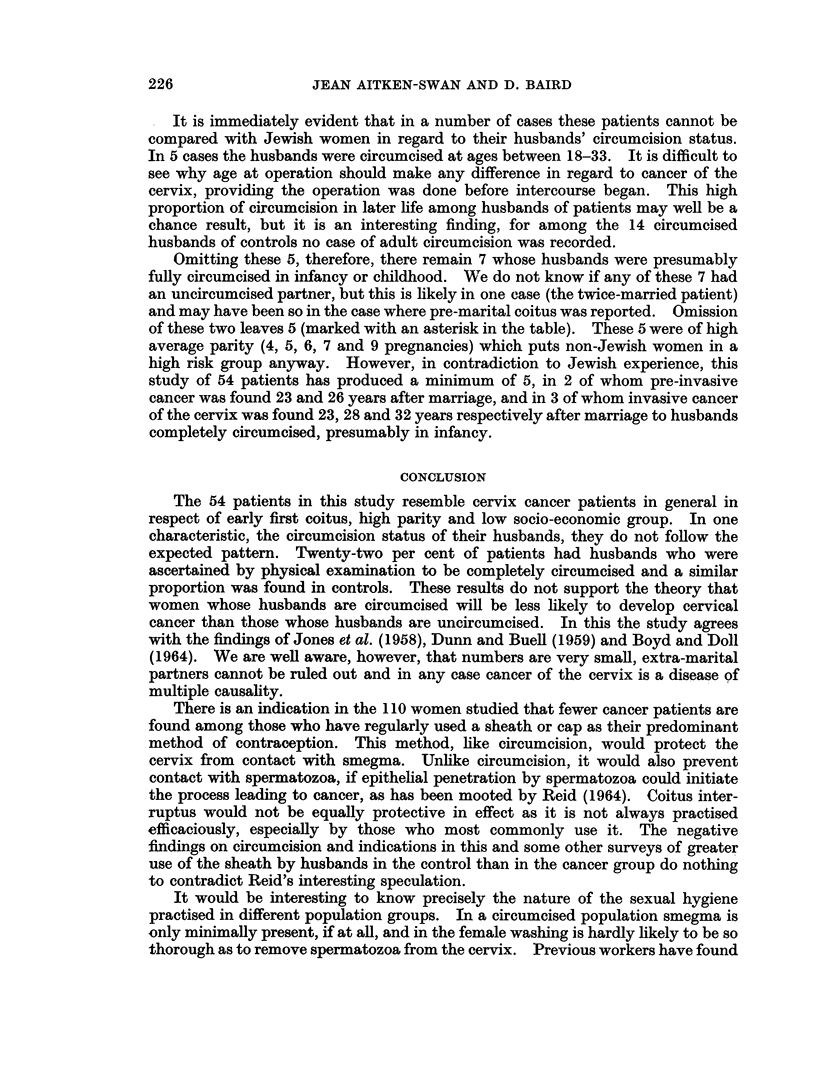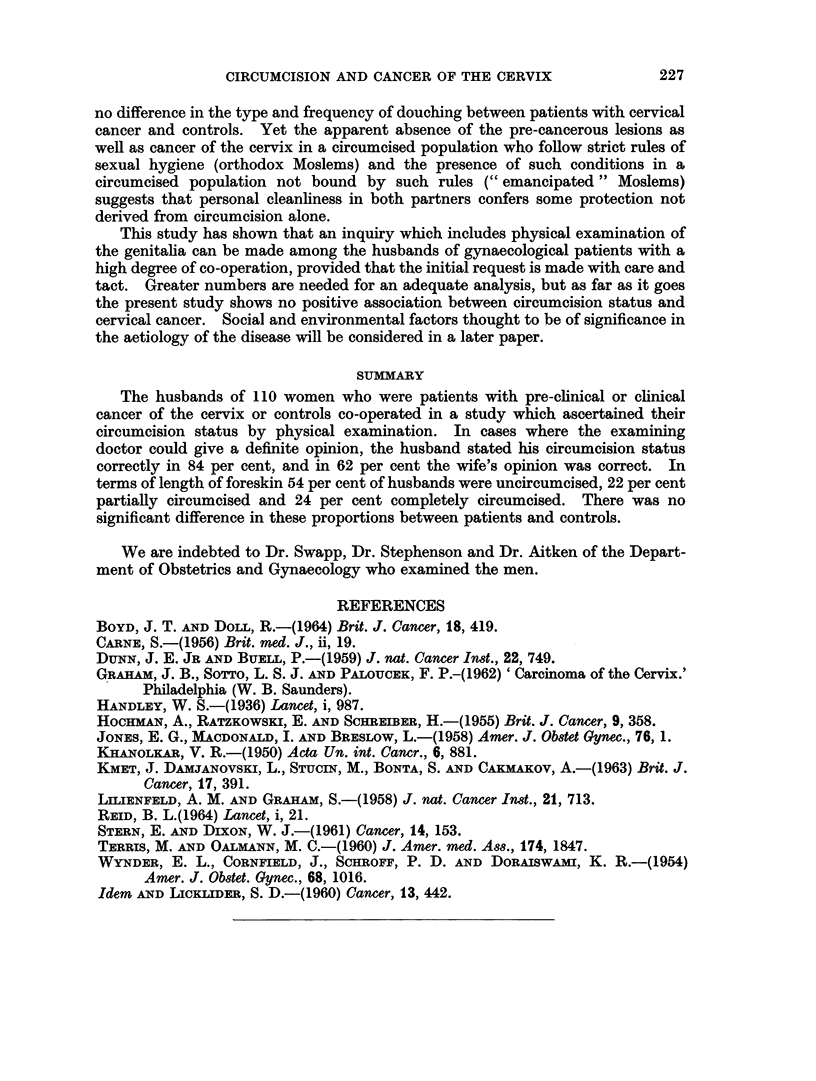# Circumcision and Cancer of the Cervix

**DOI:** 10.1038/bjc.1965.27

**Published:** 1965-06

**Authors:** Jean Aitken-Swan, D. Baird


### BRITISH JOURNAL OF CANCER

VOL. XIX JUNE, 1965 NO. 2

### CIRCUMCISION AND CANCER OF THE CERVIX

JEAN AITKEN-SWAN AND D. BAIRD

*From the Department of Obstetrics and Gynaecology, University of Aberdeen, and Obstetric Medicine Research Unit, (M.R.C.), Aberdeen*

Received for publication January 18, 1965

THE relative infrequency of cancer of the uterine cervix in Jewish women compared with women of other races has interested epidemiologists since the beginning of the century, but has not yet been explained. The average annual number of cases per 100,000 Jewish women in Israel over a 19-year period has been shown by Hochman, Ratzkowski and Schreiber (1955) to be 2·2, while the rate per 100,000 women elsewhere ranges from 17 in Sweden to 44 in a group of 10 cities in the U.S.A. Reasons for the disparity have been sought in genetics, in the Jewish ritual laws governing sexual hygiene and in the possible protective effects of universal male circumcision. Support for the circumcision hypothesis is found in the lower frequency of cancer of the cervix in other populations who practise circumcision when compared with populations who do not. Again, penile cancer is rare in those circumcised at an early age, suggesting some carcinogenic property of smegma and an association between poor penile hygiene and cancer of the cervix. It is not easy to isolate the effect of circumcision from that of many simultaneous variables and environmental differences between population groups. Circumcision has been included among the factors considered in several careful studies of the aetiology of this disease, but its importance is still in doubt. The difficulty in finding out if there is a lower rate of cancer of the cervix among wives of circumcised non-Jewish men lies in ascertaining accurately who is or is not effectively circumcised, and in obtaining the information for all of the sexual partners of each woman. While the second difficulty seems insurmountable, the present study attempts to compare, by physical examination in 110 cases, the circumcision status of present husbands of women with pre-clinical and clinically diagnosed cancer of the cervix and husbands of matched controls. Even such a small number is of interest when the facts about circumcision are definitely established. Moreover, the study has shown what degree of co-operation can be obtained in such an inquiry and what error there would be were examination dispensed with and the husband's or wife's report relied on.

### PREVIOUS WORK

In population groups other than Jewish, practising circumcision, lower rates of cervical cancer have been noted when compared with groups in the same general environment who do not practise circumcision. Examples have come from the

Fiji islands (Handley, 1936), India (Wynder *et al.*, 1954) and more recently Macedonia (Kmet *et al.*, 1963). Graham, Sotto and Paloucek (1962) point out that Handley was comparing the rate of cervical cancer in a Fijian circumcised population in whom the incidence of cancer generally was very low (Polynesian and Melanesian) with the rate in an uncircumcised immigrant Indian population, predominantly Hindu, in whom the incidence of cancer of the cervix, in India at any rate, was high. Khanolkar's (1950) figures for patients attending the Tata Memorial Hospital in Bombay, suggest a higher relative frequency of cervical cancer in Moslems than in Parsees, although the former practise circumcision and the latter do not. Parsees attach great importance to personal cleanliness, and on the assumption that this explains the low cancer frequency one would expect the Moslems to achieve the same effect by circumcision. That circumcision does not fully explain differences in rates is also indicated in Kmet's study in Macedonia. While cancer of the cervix was found less frequently in circumcised Moslems than in the uncircumcised non-Moslems, the frequency of pre-malignant and malignant conditions of the cervix was 5·5 per 1,000 in " emancipated " Moslems and nil in the orthodox Moslems adhering strictly to religious rules regarding sexual hygiene.

Wynder *et al.* (1954), in their study of environmental factors in cancer of the cervix, asked women in several hospitals whether or not their husbands were circumcised. If they did not know they were asked to find out from their husbands. Wynder admitted that these data were not ideal—the woman's or husband's statement was uncorroborated and not all could give the necessary information. They found that more husbands in the control than in the cancer group were reported to be circumcised and this was so for all the population groups studied, white non-Jewish, Negro, small group of Jewish, patients at the Memorial Clinic separately and at all clinics combined.

In a study made in Los Angeles, Jones, Macdonald and Breslow (1958), who also obtained their information by interview, found that when Jewish women were excluded circumcision was as frequent in cases as in controls. They do not discuss the validity of their circumcision data, although they regrouped cases and controls by various techniques to verify the initial conclusion.

Dunn and Buell (1959) looked critically at the contradictory findings on circumcision of these two studies. Using the material of Jones and associates, they worked out the age-standardised expected number of patients with circumcised and uncircumcised husbands. They too found no association between cervical cancer and lack of circumcision of the husband.

Further negative evidence has recently been provided by Boyd and Doll (1964) who asked married women in a survey if their husbands were circumcised. They found that a third could not give a definite answer and of the remainder very similar proportions of patients and controls said they had at one time been married to an uncircumcised man.

Contradictory results on the association between cervical cancer and circumcision raised doubts about the diagnostic criteria of circumcision used and the validity of statements by the patients. Noting Wynder's difficulty in obtaining reliable facts about the husbands' circumcision status, Lilienfeld and Graham (1958) compared statements of 192 male patients on their circumcision status with the findings of the physician making the first routine physical examination of the patient on admission to hospital. They found disagreement between statement and examination results in about 34 per cent of cases, most frequently in those who said that they were not circumcised when physical examination indicated the reverse. They found no explanation of the wide discrepancy in unfamiliarity with the English language, lack of education (as indicated by occupation) or in mistakes in classification by one or more of the examining physicians. They considered that it was due to differences in the criteria of circumcision used by the physicians. The fact that surgical circumcision had been performed was of less importance than the actual amount of foreskin removed.

Dunn and Buell (1959) comparing the opinions of 184 men on their circumcision status with the results of an examination made by physicians, decided that the only question that could be answered definitely by the examination was : is the patient effectively circumcised anatomically? They found that over a third of the men (37·5 per cent) were partially circumcised, suggesting that a sizeable proportion of non-Jewish males in the U.S.A. have a short foreskin which occurs naturally or as a result of operation. By contrast, Jewish men were almost invariably fully circumcised.

In a further study to test the reliability of circumcision data Wynder and Licklider (1960) interviewed and examined 100 male hospital patients in New York and a similar number in Los Angeles, care being taken to use the same criteria defining circumcision. A quarter of the 200 male patients were unable to state correctly their circumcision status. They also asked a similar number of female patients in the two cities for the circumcision status of their husbands, and the proportion thought to be circumcised did not correspond with the proportion found in the 200 men examined. They conclude that to find out whether circumcision has or has not been performed physical examination is mandatory and also that where the foreskin is present its length is probably of great practical importance.

### PRESENT STUDY

An opportunity to take the matter further was provided by a study of social and environmental factors in cancer of the cervix in Aberdeen. Women in three categories were being interviewed, those with clinically diagnosed squamous cell cancer of the cervix, those with cancer not diagnosable clinically but by cytology and subsequent histology, and matched controls for the latter, chosen from among those with negative smears. The interview with the woman being completed, a letter with stamped, addressed envelope was left for her husband, inviting him to assist in the research by contributing some information that he alone could give, involving a visit to the hospital out of working hours and a brief examination by a doctor. Rather surprisingly, as many as 47 per cent of the husbands came for interview.

TABLE I.—*Success Rate according to Wife's Category*

| | Asked | Came | % |
|---|---|---|---|
| Clinical | 17 | 10 | 59 |
| Pre-clinical | 92 | 44 | 48 |
| Control | 126 | 56 | 44 |
| | 235 | 110 | 47 |

Equal proportions in each of 3 occupational class groups co-operated. Husbands of the women in the clinically diagnosed group were the most co-operative although the total numbers were small because of the high proportion of widowed, divorced or separated women in this category. The " controls " in this study do not necessarily match the pre-clinical group due to self-selection, in that only certain individuals responded to the request to take part.

The object of the study was to investigate :

1. Whether the wife's statement on her husband's circumcision status tallied with the doctor's opinion,
2. to what extent the husband's statement tallied with the doctor's opinion, and
3. regardless of surgical circumcision, did the foreskin
   (a) completely cover the glans,
   (b) partly cover the glans, or
   (c) was it completely absent?

Was there any difference in this respect between husbands of women with pre-clinical and clinical lesions on the one hand and controls on the other?

About 60 per cent of the men were seen by one Registrar from the Department of Obstetrics and Gynaecology, about a quarter by another and the remainder by other Registrars as available. All recorded the same type of information on a standard form.

### RESULTS

### *Wife's statement on whether husband circumcised*

In 5 instances the husband was present and answered for himself. Thirty-three wives said they did not know. Seventy-two stated their opinion, which proved to be right in 60, wrong in 6 and in 6 the doctor could not be sure even after examination.

Where the wife was *certain* she knew (36 cases) she was right in 34 and could have been right in the other 2 where the doctor was not sure. When she only *thought* she knew (36 cases) she was right in 26, wrong in 6 and the doctor was not sure in the other 4.

TABLE II.—*Wife's Statement by Doctor's Findings*

| | Doctor's opinion | | |
|---|---|---|---|
| Wife reports | Circumcised | Uncircumcised | Cannot be sure |
| Circumcised | 13 | 0 | 1 |
| Uncircumcised | 6 | 47 | 5 |
| Does not know | 4 | 26 | 3 |
| Not asked | 1 | 4 | 0 |
| Total | 24 | 77 | 9 |

### *Husband's statement on his circumcision status*

There was agreement between the husband's statement and the doctor's findings in 85 of the 110 cases. In 5 there was disagreement, and in the remaining 20 either the man did not know or the doctor could not be sure if an operation had been done.

Table IV shows the husband's statement in relation to the physical findings. An ambiguous group is the intermediate category (b) with partial lack of foreskin.

TABLE III.—*Husband's Statement by Doctor's Findings*

| Husband reports | Doctor's opinion: Circumcised | Uncircumcised | Cannot be sure |
|---|---|---|---|
| Circumcised | 19 | 1 | 3 |
| Uncircumcised | 4 | 66 | 6 |
| Does not know | 1 | 10 | 0 |
| Total | 24 | 77 | 9 |

In at least 17 of these 24 cases the doctor considered this to be a natural effect, but in another 6 he could not be sure. In only one was the effect attributed to incomplete operation. Reliance on the husband's statement would have relegated most of these cases to the uncircumcised group, but it is possible that partial circumcision affords partial protection against the risk of cancer, and this category is shown separately in the tables that follow.

TABLE IV.—*Husband's Statement by Physical Findings*

| Physical findings | Husband reports: Circumcised | Uncircumcised | Does not know |
|---|---|---|---|
| (a) Foreskin completely covers the glans | 1 | 52 | 7 |
| (b) Foreskin partly covers the glans | 4 | 17 | 3 |
| (c) Foreskin completely absent | 18 | 7 | 1 |
| Total | 23 | 76 | 11 |

*Husband's circumcision status, surgical or natural effect*

In 60 of the 110 cases the husbands were uncircumcised (a), in 24 partially circumcised (b) and in 26 completely circumcised (c), in terms of length of foreskin. The small numbers show no significant difference in these categories between pre-clinical, clinical cases and controls.

TABLE V.—*Husband's Circumcision Status, Surgical or Natural, Determined by Physical Examination.*

| Physical findings | Pre-clinical No. | Clinical No. | Pre-clinical and clinical combined Total | % | Controls No. | % |
|---|---|---|---|---|---|---|
| (a) Foreskin completely covers the glans | 28 | 4 | 32 | 59 | 28 | 50 |
| (b) Foreskin partly covers the glans | 8 | 2 | 10 | 19 | 14 | 25 |
| (c) Foreskin completely absent | 8 | 4 | 12 | 22 | 14 | 25 |
| Total | 44 | 10 | 54 | 100 | 56 | 100 |

Contrary to Jewish experience, it appears that the small group of 54 husbands of women with pre-clinical and clinical cancer has included 12 who were completely circumcised (c). The interview with the wife supplies some background information about this interesting group and Table VI compares some of their characteristics with those of cancer patients and controls whose husbands were uncircumcised or partially circumcised.

TABLE VI.—*Characteristics of Women by Circumcision Status of Husband**

| Characteristics | Pre-clinical and clinical Total No. | % | Un-Circ. (a) No. | Partly Circ. (b) No. | Circ. (c) No. | Controls Total No. | % | Un-Circ. (a) No. | Partly Circ. (b) No. | Circ. (c) No. |
|---|---|---|---|---|---|---|---|---|---|---|
| (a) *Age Group* | | | | | | | | | | |
| 25– | 15 | 28 | 11 | 1 | 3 | 26 | 46 | 14 | 6 | 6 |
| 35– | 20 | 37 | 11 | 5 | 4 | 17 | 30 | 6 | 5 | 6 |
| 45– | 13 | 24 | 6 | 4 | 3 | 11 | 20 | 7 | 2 | 2 |
| 55– | 4 | 7 | 2 | — | 2 | 1 | 2 | — | 1 | — |
| 65+ | 2 | 4 | 2 | — | — | 1 | 2 | 1 | — | — |
| (b) *Number of marriages* | | | | | | | | | | |
| One | 50 | 93 | 31 | 8 | 11 | 55 | 98 | 14 | 13 | 28 |
| Two | 4 | 7 | 1 | 2 | 1 | 1 | 2 | — | 1 | — |
| (c) *Age at first coitus* | | | | | | | | | | |
| <20 | 21 | 39 | 13 | 4 | 4 | 18 | 32 | 7 | 3 | 8 |
| 20–24 | 21 | 39 | 11 | 4 | 6 | 28 | 50 | 15 | 9 | 4 |
| 25+ | 11 | 20 | 7 | 2 | 2 | 10 | 18 | 6 | 2 | 2 |
| Not stated | 1 | 2 | 1 | — | — | — | — | — | — | — |
| (d) *Number of pregnancies* | | | | | | | | | | |
| 0 | 1 | 2 | — | 1 | — | — | — | — | — | — |
| 1, 2 | 16 | 30 | 10 | 4 | 2 | 18 | 32 | 11 | 4 | 3 |
| 3, 4 | 22 | 40 | 14 | 4 | 4 | 29 | 52 | 14 | 7 | 8 |
| 5+ | 15 | 28 | 8 | 1 | 6 | 9 | 16 | 3 | 3 | 3 |
| (e) *Occupational group* | | | | | | | | | | |
| Non-manual | 6 | 11 | 5 | 1 | — | 17 | 30 | 11 | 2 | 4 |
| Skilled manual | 20 | 37 | 11 | 6 | 3 | 21 | 38 | 8 | 7 | 6 |
| Semi- and unskilled manual | 28 | 52 | 16 | 3 | 9 | 18 | 32 | 9 | 5 | 4 |
| (f) *Predominant method of contraception* | | | | | | | | | | |
| Sheath | 1 | 2 | 1 | — | — | 14 | 25 | 8 | 4 | 2 |
| Cap | 4 | 7 | 4 | — | — | 6 | 11 | 3 | — | 3 |
| Both | 2 | 4 | 1 | 1 | — | 4 | 7 | 2 | — | 2 |
| Coitus interruptus | 22 | 41 | 13 | 4 | 5 | 15 | 27 | 8 | 4 | 3 |
| Other | 3 | 6 | 2 | 1 | — | 2 | 4 | — | 1 | 1 |
| No predominant method | 11 | 20 | 6 | 1 | 4 | 14 | 25 | 7 | 4 | 3 |
| No contraception | 11 | 20 | 5 | 3 | 3 | 1 | 2 | — | 1 | — |
| (g) *Interval first coitus to first attendance at hospital* | | | | | | | | | | |
| 5–14 years | 18 | 33 | 13 | 2 | 3 | — | — | — | — | — |
| 15–24 years | 22 | 41 | 12 | 6 | 4 | — | — | — | — | — |
| 25 years + | 14 | 26 | 7 | 2 | 5 | — | — | — | — | — |
| Totals | 54 | 100 | 32 | 10 | 12 | 56 | 100 | 28 | 14 | 14 |

* The 3 circumcision categories here refer to length of foreskin, as in Table V and not to surgical circumcision only.

The totals column for 4 of the first 5 variables confirms what is already known about cervical cancer patients compared with the female population as a whole.

More of them are twice married, start coitus at an early age, they have larger families and fewer are married to men in non-manual occupations. Most of these characteristics are independent of the circumcision status of the husband, but the numbers circumcised may be affected by age and occupational class.

*Age and circumcision*

Assuming that the husbands are in the same 15-year age group as their wives, there is little difference between 3 age groups, 25–39, 40–54 and 55 and over in the proportion completely circumcised. Little is known of the circumcision status of the population, but a check made in 1960 among 83 surgical ward patients, also Aberdeen city residents, showed a lower overall rate of circumcision, (17 per cent fully circumcised compared with 24 per cent in the present survey). In the 2 surveys the proportion circumcised was not significantly different in the younger age groups. At age 55–69 a higher proportion of circumcised is found in the present survey but numbers are small for comparison.

*Occupational class and circumcision*

Available data suggest that circumcision is more common in the higher socio-economic groups. Carne (1956) in his study of R.A.F. recruits aged 17–24 found different rates of circumcision according to the type of school the recruits had attended. Rates for the United Kingdom as a whole were highest for those from public schools (41·9 per cent), lower for those from grammar schools (36·6 per cent) and lowest for those from elementary schools (32·8 per cent). He found that rates were uniformly lower in Scotland than in England (35·4 to 20 per cent circumcised). Table VI (e) shows the proportion completely circumcised to range from 18 per cent of the non-manual to 30 per cent of the unskilled manual group, but numbers are too small for this to have any particular significance.

*Contraceptives and circumcision*

If penile cleanliness protects the cervix from cancer, circumcision is not the only means of attaining this. Good standards of personal hygiene should prevent the formation of smegma equally effectively. Protection might also be afforded by the regular use of a contraceptive, such as a sheath or cap. Stern and Dixon (1961), considering the prevalence of dysplasia, *in situ* cancer and invasive cancer of the cervix in women attending a cancer detection centre, studied several variables thought to be of significance in the aetiology of cervical cancer by methods of multiple regression analysis. They found both circumcision and contraception to be of secondary significance. Terris and Oalmann (1960) in an epidemiological study of patients with cervical cancer and controls, homogeneous in regard to educational level, religion and husband's or father's occupation, found that few used contraceptives (mostly sheath or cap), but that the proportion doing so was significantly lower in patients than in controls. Boyd and Doll (1964) considering different methods of contraception ever used by patients and controls found that the use of an obstructive method by either husband or wife was less frequent in patients than in controls.

The present study agrees with that of Boyd and Doll in finding that fewer patients than controls had " ever used " or " predominantly used " a sheath or cap. Thirteen per cent of patients reported the use of sheath or cap as their predominant or main regular method of contraception compared with 43 per cent of controls (Table VI f). The difference is partly accounted for by the preponderance in the control group of non-manual workers, the main regular users of the sheath or cap (Table VII). However, the proportion using a sheath or cap as their predominant method of contraception is higher among controls than patients in each occupational group separately.

TABLE VII.—*Predominant Method of Contraception by Occupational Group*

| Method of contraception | Non-manual N=23 | Skilled N=41 | Semi- and Unskilled N=46 |
|---|---|---|---|
| | % | % | % |
| Sheath, cap or both | 61 | 24 | 15 |
| Coitus interruptus | 26 | 44 | 28 |
| Other | — | 5 | 7 |
| No predominant method | 13 | 22 | 28 |
| No contraception | — | 5 | 22 |
| Total | 100 | 100 | 100 |

Looked at in another way, Table VIII shows the total numbers in the survey regularly using or not using sheath or cap contraception. The proportion of patients in this total is shown for the 3 circumcision categories separately.

TABLE VIII.—*Proportion of Patients Among Regular Users and Non-users of Sheath or Cap Contraception*

| Circumcision status of husband | Using sheath or cap regularly | | | Not using sheath or cap regularly | | |
|---|---|---|---|---|---|---|
| | | Patients | | | Patients | |
| | Total | No. | % | Total | No. | % |
| Uncircumcised (a) | 19 | 6 | 31 | 41 | 26 | 63 |
| Partly circumcised (b) | 5 | 1 | 20 | 19 | 9 | 47 |
| Circumcised (c) | 7 | 0 | 0 | 19 | 12 | 63 |
| Total | 31 | 7 | 22 | 79 | 47 | 59 |

The bottom line shows that when the husband was circumcised and the couple regularly used a sheath or cap (7 cases) there were no patients. Where the husband was circumcised but there was no regular use of sheath or cap (19 cases) 12 were patients. The other two circumcision categories show the same trend—fewer patients where there is regular use of sheath or cap. Clearly numbers are much too small for firm conclusions to be reached, but Table VIII suggests that circumcision helps if a sheath or cap is regularly used as well. If the women's statement is correct, she has complete protection at all times from contact with smegma. Where the husband is partly circumcised or uncircumcised, she does not have complete protection, despite the regular use of sheath or cap.

*Interval from first coitus to first attendance at hospital : patients*

Assuming first coitus to be the stimulus initiating the process leading to the finding of cancer, how is the interval between the two events affected by circumcision or by the regular use of sheath or cap contraception? If these measures prevent the occurrence of cancer in some, they may also delay its onset in others.

Table VI (g) shows the interval from first coitus to first attendance at the hospital for patients with husbands in the 3 circumcision categories. Although the difference between the fully circumcised and fully uncircumcised groups is in the direction of a longer interval where the husband is fully circumcised the numbers are obviously too small for significance. The difference disappears if those regularly using a sheath or cap are added to the number with husband fully circumcised (that is, women with presumed maximum protection) and compared with those not regularly using these methods and with husband fully uncircumcised (minimum protection) (Table IX).

TABLE IX.—*Interval from First Coitus to First Attendance at Hospital in Patients with (a) Maximum Protection and (b) Minimum Protection*

| Interval | Maximum protection No. | Maximum protection % | Minimum protection No. | Minimum protection % |
|---|---|---|---|---|
| Under 15 years | 7 | 37 | 9 | 35 |
| 15–24 years | 7 | 37 | 10 | 38 |
| 25 years and over | 5 | 26 | 7 | 27 |
| Total | 19 | 100 | 26 | 100 |

*Women with pre-clinical or clinical cancer of the cervix whose husbands are fully circumcised*

The study has shown (Table V) that complete circumcision is found about as frequently in husbands of patients with cancer of the cervix as in husbands of controls. As the patient group is of particular interest and the numbers small, the 12 with fully circumcised husbands are set out individually in Table X.

TABLE X.—*Patients with Husband Completely Circumcised*

| Age at 1st coitus | Interval 1st coitus to 1st attendance at hospital | No. of pregnancies | When husband circumcised | Remarks | Type of cancer |
|---|---|---|---|---|---|
| | | | *Pre-clinical* | | |
| 17 | 14 | 3 | 26 (year of marriage) | Coitus 4 years before marriage | Invasive |
| 18 | 17 | 5 | Says not | Twice married | Pre-invasive |
| 18* | 26 | 7 | Says not | | Pre-invasive |
| 19* | 23 | 4 | At 1 year | | Pre-invasive |
| 20 | 8 | 3 | Infancy | Coitus 1 year before marriage | |
| 20* | 23 | 6 | Infancy | | Early invasive |
| 22 | 10 | 4 | 28—single | For balanitis | Pre-invasive |
| 24 | 23 | 2 | 33—married at 27 | Recurrent balanitis and paraphimosis | Invasive |
| | | | *Clinical* | | |
| 20* | 32 | 9 | Says not | | Invasive |
| 22* | 28 | 5 | Does not recall | | Invasive |
| 25 | 34 | 9 | 18 (before marriage) | | Invasive |
| 27 | 36 | 2 | 21 (before marriage) | Had jaundice at the time | Invasive |

It is immediately evident that in a number of cases these patients cannot be compared with Jewish women in regard to their husbands' circumcision status. In 5 cases the husbands were circumcised at ages between 18–33. It is difficult to see why age at operation should make any difference in regard to cancer of the cervix, providing the operation was done before intercourse began. This high proportion of circumcision in later life among husbands of patients may well be a chance result, but it is an interesting finding, for among the 14 circumcised husbands of controls no case of adult circumcision was recorded.

Omitting these 5, therefore, there remain 7 whose husbands were presumably fully circumcised in infancy or childhood. We do not know if any of these 7 had an uncircumcised partner, but this is likely in one case (the twice-married patient) and may have been so in the case where pre-marital coitus was reported. Omission of these two leaves 5 (marked with an asterisk in the table). These 5 were of high average parity (4, 5, 6, 7 and 9 pregnancies) which puts non-Jewish women in a high risk group anyway. However, in contradiction to Jewish experience, this study of 54 patients has produced a minimum of 5, in 2 of whom pre-invasive cancer was found 23 and 26 years after marriage, and in 3 of whom invasive cancer of the cervix was found 23, 28 and 32 years respectively after marriage to husbands completely circumcised, presumably in infancy.

### CONCLUSION

The 54 patients in this study resemble cervix cancer patients in general in respect of early first coitus, high parity and low socio-economic group. In one characteristic, the circumcision status of their husbands, they do not follow the expected pattern. Twenty-two per cent of patients had husbands who were ascertained by physical examination to be completely circumcised and a similar proportion was found in controls. These results do not support the theory that women whose husbands are circumcised will be less likely to develop cervical cancer than those whose husbands are uncircumcised. In this the study agrees with the findings of Jones *et al.* (1958), Dunn and Buell (1959) and Boyd and Doll (1964). We are well aware, however, that numbers are very small, extra-marital partners cannot be ruled out and in any case cancer of the cervix is a disease of multiple causality.

There is an indication in the 110 women studied that fewer cancer patients are found among those who have regularly used a sheath or cap as their predominant method of contraception. This method, like circumcision, would protect the cervix from contact with smegma. Unlike circumcision, it would also prevent contact with spermatozoa, if epithelial penetration by spermatozoa could initiate the process leading to cancer, as has been mooted by Reid (1964). Coitus interruptus would not be equally protective in effect as it is not always practised efficaciously, especially by those who most commonly use it. The negative findings on circumcision and indications in this and some other surveys of greater use of the sheath by husbands in the control than in the cancer group do nothing to contradict Reid's interesting speculation.

It would be interesting to know precisely the nature of the sexual hygiene practised in different population groups. In a circumcised population smegma is only minimally present, if at all, and in the female washing is hardly likely to be so thorough as to remove spermatozoa from the cervix. Previous workers have found no difference in the type and frequency of douching between patients with cervical cancer and controls. Yet the apparent absence of the pre-cancerous lesions as well as cancer of the cervix in a circumcised population who follow strict rules of sexual hygiene (orthodox Moslems) and the presence of such conditions in a circumcised population not bound by such rules ("emancipated" Moslems) suggests that personal cleanliness in both partners confers some protection not derived from circumcision alone.

This study has shown that an inquiry which includes physical examination of the genitalia can be made among the husbands of gynaecological patients with a high degree of co-operation, provided that the initial request is made with care and tact. Greater numbers are needed for an adequate analysis, but as far as it goes the present study shows no positive association between circumcision status and cervical cancer. Social and environmental factors thought to be of significance in the aetiology of the disease will be considered in a later paper.

SUMMARY

The husbands of 110 women who were patients with pre-clinical or clinical cancer of the cervix or controls co-operated in a study which ascertained their circumcision status by physical examination. In cases where the examining doctor could give a definite opinion, the husband stated his circumcision status correctly in 84 per cent, and in 62 per cent the wife's opinion was correct. In terms of length of foreskin 54 per cent of husbands were uncircumcised, 22 per cent partially circumcised and 24 per cent completely circumcised. There was no significant difference in these proportions between patients and controls.

We are indebted to Dr. Swapp, Dr. Stephenson and Dr. Aitken of the Department of Obstetrics and Gynaecology who examined the men.

REFERENCES

BOYD, J. T. AND DOLL, R.—(1964) *Brit. J. Cancer*, **18**, 419.
CARNE, S.—(1956) *Brit. med. J.*, ii, 19.
DUNN, J. E. JR AND BUELL, P.—(1959) *J. nat. Cancer Inst.*, **22**, 749.
GRAHAM, J. B., SOTTO, L. S. J. AND PALOUCEK, F. P.–(1962) 'Carcinoma of the Cervix.' Philadelphia (W. B. Saunders).
HANDLEY, W. S.—(1936) *Lancet*, i, 987.
HOCHMAN, A., RATZKOWSKI, E. AND SCHREIBER, H.—(1955) *Brit. J. Cancer*, **9**, 358.
JONES, E. G., MACDONALD, I. AND BRESLOW, L.—(1958) *Amer. J. Obstet Gynec.*, **76**, 1.
KHANOLKAR, V. R.—(1950) *Acta Un. int. Cancr.*, **6**, 881.
KMET, J. DAMJANOVSKI, L., STUCIN, M., BONTA, S. AND CAKMAKOV, A.—(1963) *Brit. J. Cancer*, **17**, 391.
LILIENFELD, A. M. AND GRAHAM, S.—(1958) *J. nat. Cancer Inst.*, **21**, 713.
REID, B. L.(1964) *Lancet*, i, 21.
STERN, E. AND DIXON, W. J.—(1961) *Cancer*, **14**, 153.
TERRIS, M. AND OALMANN, M. C.—(1960) *J. Amer. med. Ass.*, **174**, 1847.
WYNDER, E. L., CORNFIELD, J., SCHROFF, P. D. AND DORAISWAMI, K. R.—(1954) *Amer. J. Obstet. Gynec.*, **68**, 1016.
*Idem* AND LICKLIDER, S. D.—(1960) *Cancer*, **13**, 442.